# Three-Dimensional Phylogeny Explorer: Distinguishing paralogs, lateral transfer, and violation of "molecular clock" assumption with 3D visualization

**DOI:** 10.1186/1471-2105-8-213

**Published:** 2007-06-20

**Authors:** Namshin Kim, Christopher Lee

**Affiliations:** 1 Center for Computational Biology, Molecular Biology Institute, Institute for Genomics and Proteomics, Department of Chemistry and Biochemistry, University of California, Los Angeles, 90095-1570, USA

## Abstract

**Background:**

Construction and interpretation of phylogenetic trees has been a major research topic for understanding the evolution of genes. Increases in sequence data and complexity are creating a need for more powerful and insightful tree visualization tools.

**Results:**

We have developed 3D Phylogeny Explorer (3DPE), a novel phylogeny tree viewer that maps trees onto three spatial axes (species on the X-axis; paralogs on Z; evolutionary distance on Y), enabling one to distinguish at a glance evolutionary features such as speciation; gene duplication and paralog evolution; lateral gene transfer; and violation of the "molecular clock" assumption. Users can input any tree on the online 3DPE, then rotate, scroll, rescale, and explore it interactively as "live" 3D views. All objects in 3DPE are clickable to display subtrees, connectivity path highlighting, sequence alignments, and gene summary views, and etc. To illustrate the value of this visualization approach for microbial genomes, we also generated 3D phylogeny analyses for all clusters from the public COG database. We constructed tree views using well-established methods and graph algorithms. We used Scientific Python to generate VRML2 3D views viewable in any web browser.

**Conclusion:**

3DPE provides a novel phylogenetic tree projection method into 3D space and its web-based implementation with live 3D features for reconstruction of phylogenetic trees of COG database.

## Background

Visual representation of phylogenetic trees is an active research topic in evolutionary biology, because it is the main way that scientists view and interpret phylogenetic trees. Traditionally, phylogenetic trees have been represented as two-dimensional (flat) diagrams [1-3]. This is not due to phylogeny being in any sense "naturally" two-dimensional, but rather to the constraints of drawing such diagrams on paper. However, as sequence datasets grow exponentially due to genome and other sequencing projects, the difficulties of interpreting phylogenetic tree diagrams are also growing in size and complexity. Recently, there have been many efforts to improve visualization of complex phylogenetic trees [3-10]. One can simplify 2D trees by merging similar subtrees into higher taxonomy classification [5]. This "data compression" procedure can make huge trees into simpler forms.

It is useful to consider how tree interpretation is limited by the assumption of two dimensionality. Since one dimension ordinarily represents the evolutionary distance metric (e.g. how distant two sequences are from their most recent common ancestor (MRCA)), the 2D assumption leaves only one dimension for representing other information. For example, in one common application ("Tree of Life", analyzing a gene sequence from different species, to determine the evolutionary relationships of these species), this second dimension is used to indicate species relationships, by placing the most closely related species proximal to each other on this dimension. However, modern gene phylogenies are far more complex than just this speciation process. Paralogous gene families, gene duplication and loss, and horizontal gene transfer are major processes of evolution that visualization should also reveal, orthogonal to speciation. Unfortunately, trying to squeeze these very different dimensions of evolution onto a single spatial axis violates a basic principle of visual data-mining, namely that each independent variable should be assigned an orthogonal axis. Combining many different variables into one can make it hard to see any of them clearly. Thus, whereas two dimensions may be adequate for "Tree of Life" phylogenies containing exactly one sequence per species (i.e. the tree consists only of speciation branching), more complex phylogenies may be obscured by the two-dimensional assumption.

An obvious solution is to apply three-dimensional visualization techniques to phylogenetic trees, so that multiple independent variables (e.g. *species *vs. *paralog *vs. *evolutionary distance*) can each be assigned an orthogonal axis. We will refer to this approach as "dimensional visualization of phylogeny" to emphasize the idea that each independent variable is viewed on a separate dimension.

Recently there has been growing interest in three-dimensional visualization of trees, for example projecting a two-dimensional tree onto a three-dimensional surface such as a disk or cone [4] that can be rotated or zoomed in 3D. We will refer to this approach as "projection of phylogeny onto a surface". Such projections can provide a useful interface for navigating a large, complex tree (using intuitive 3D operations such as rotation). However, this is quite different from dimensional visualization of phylogeny. In the projection approach the tree is still visualized on a *surface *(the two dimensional surface of a three dimensional disk or cone), and the layout of the tree on this 2D surface does not differ fundamentally from standard 2D tree layout.

**Figure 1:**
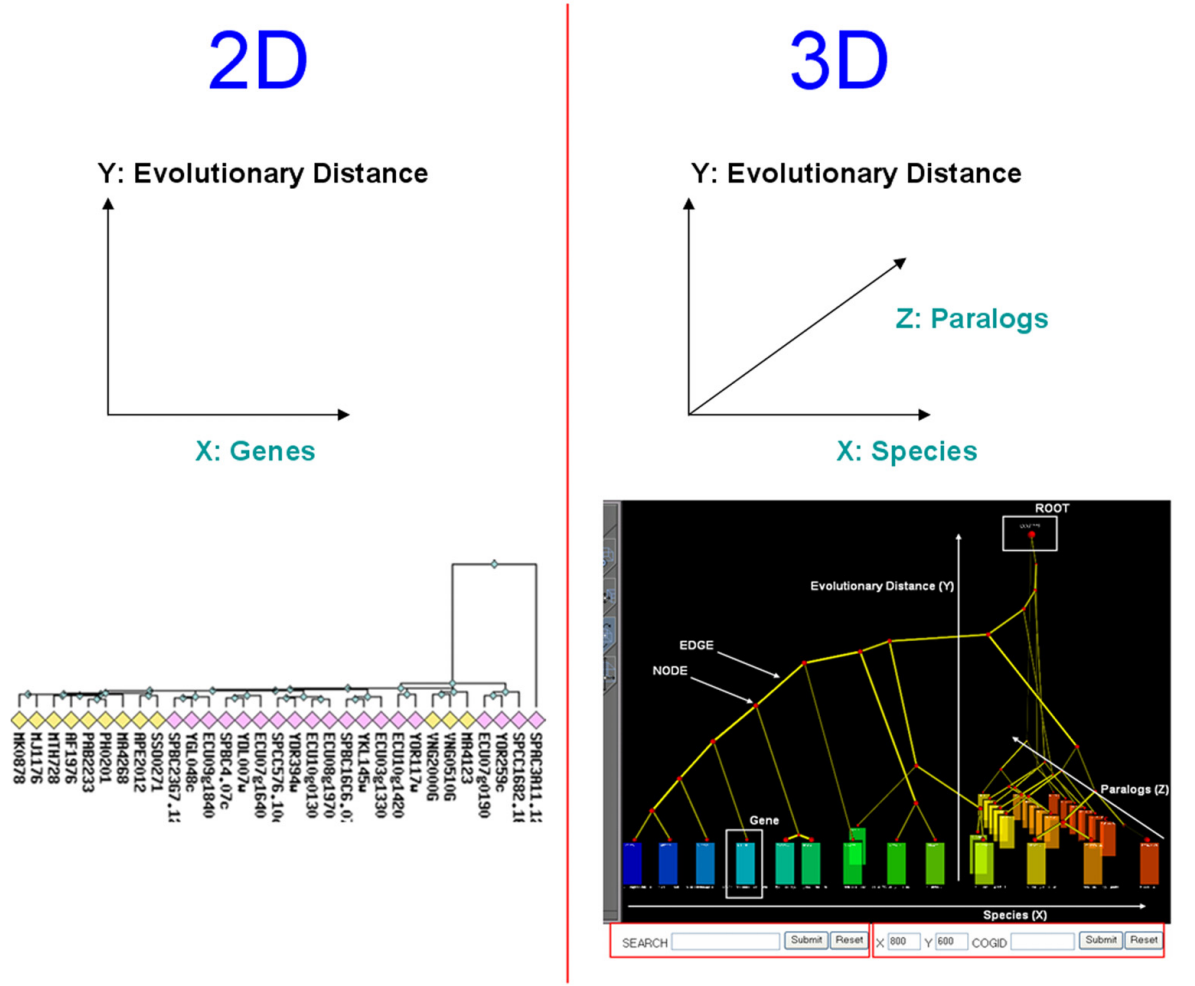
**2D vs. 3DPE views of the same tree**. In standard (2D) phylogeny layout it is not always easy to distinguish gene duplication events (paralogs) from speciation branching (species), because only one spatial axis (labelled "genes" in the diagram above) is available to show the mix of these two kinds of information. By contrast, they can be distinguished at a glance in 3DPE, because it projects them onto two orthogonal axes; species (X) vs. paralogs (Z). For example, whereas the evolution of a large number of paralogs is visually obvious in the 3DPE view (in the three eukaryote species, on the right), this pattern is less clear in the 2D representation. Both trees represent COG1222, and 2D tree was downloaded from the COG database.

In this paper we describe 3-Dimensional Phylogeny Explorer (3DPE), a novel algorithm for phylogeny layout that implements the dimensional visualization of phylogeny approach. It maps trees onto three spatial axes (*species *on the X-axis; *paralogs *on Z; *evolutionary distance *on Y), making interpretation of the tree visually intuitive (Fig. 1). Sequences with the same X coordinate come from the same species (and therefore must be paralogs). Sequences with the same Z coordinate constitute one orthologous group (i.e. they are orthologs, the "same gene" in different species). As is conventional for phylogenetic trees, the Y coordinate (height) represents evolutionary distance (often treated as a proxy for evolutionary time). Using this dimensional visualization, it is easy to distinguish at a glance different events such as speciation, gene duplication and horizontal gene transfer. We have created a web service that creates 3D visualizations of user-supplied phylogenetic trees viewable in any VRML-enabled web browser or VRML viewer. Furthermore, to illustrate the utility of this visualization approach, we have applied it to the well-known COG database, a large-scale database of gene families from microbial genomes [11-14]. Using 3DPE, one can easily navigate phylogenetic trees in 'real' 3D space as well as interactive online tree manipulations.

One important aspect of 3DPE is that users can employ any operational taxonomic unit (OTU) choice that they wish to display on 3DPE's X-axis, by supplying whatever OTU information they like in the user-supplied tree format. Because the simple examples shown in this paper are all based on *species *as the OTU (using species information from the COG database), we will refer to the OTU in these examples as "species". As defined by Koonin, "Orthologs are genes derived from a single ancestral gene in the last common ancestor of the compared species, and paralogs are genes related via duplication [15]". We follow this definition in 3DPE and in this paper.

## Methods

### Introduction to 3DPE tree layout

3DPE differs from traditional 2D tree layout in several ways. One can consider 2D phylogenetic trees to represent two distinct variables: *sequences *on the X-axis and *evolutionary distance *on Y. (e.g. for a Tree of Life phylogeny, there is a 1:1 mapping between individual *sequences *and individual *species*, because there should only be a single sequence for each species). A given 2D tree can have many possible ordering of sequences from left to right (a tree with N internal branch nodes has 2^N ^possible orderings) that are equally valid. By contrast, 3DPE projects phylogenetic trees onto three spatial axes as shown in Figure 1. The core operation in 3DPE is that leaves (sequences) must be assigned (X, Z) coordinates (*species *on the X-axis; *paralogs *on Z); the Y coordinate represents *evolutionary distance*, just as in conventional 2D trees. Specifically, every *species *must be assigned a distinct X value; every *sequence *must be assigned to one *orthologous group*; and each *orthologous group *must be assigned a distinct Z value.

### Source data: user phylogenetic trees

3DPE takes a standard phylogenetic tree as the starting point of its analysis. Currently, 3DPE can take a user-supplied phylogenetic tree in the Newick format, or phylogenetic tree files from PHYLIP. One important point is that 3DPE needs species information for all sequences in the user-supplied tree, i.e. each species must be assigned a unique identifier, and each sequence must have a species attribute giving the ID of its source species. 3DPE follows a simple convention for reading species information from a sequence "name" string: the sequence name string must be of the form '*species|gene*' (e.g. "Mac|MA4123" means "Mac" is the species identifier, and "MA4123" is the gene identifier). 3DPE requires that the user-supplied tree provide branch length information, indicating the evolutionary distance between each pair of nodes.

### Defining orthologous groups

**Figure 2:**
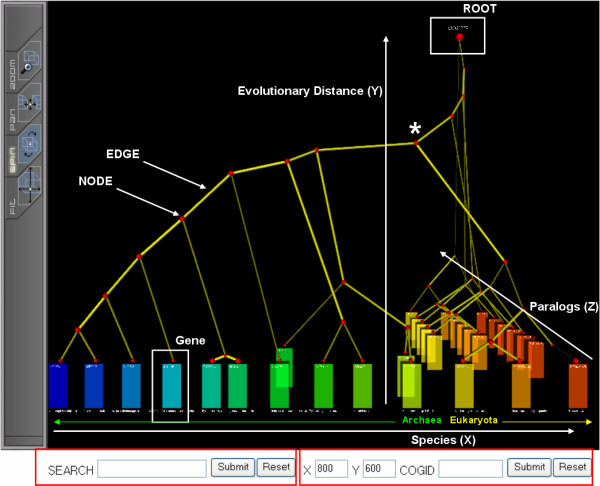
**3DPE main view mode for COG1222, ATP-dependent 26S proteasome regulatory subunit**. 3DPE projects evolutionary information onto three spatial axes: each species is mapped to a unique location of on the X-axis; each orthologous group is assigned a unique plane on the Z-axis; and the Y-axis represents evolutionary distance. This can both yield valuable insights and reveal potential problems with the tree; e.g. the absence of paralogs in the Archaea suggests that these paralogs were created after the divergence of Eukaryota and Archaea, and thus that the tree's root should actually be at the node marked with an asterisk (*). Thus, 3DPE can make it easier to spot potential errors in the tree, which could be tested using other methods such as outgroup analysis. Genes are represented as hexagonal boxes and each species has its distinct color. Species (Taxonomy IDs): 2234, 145262, 190192, 2190, 29292, 53953, 188937, 56636, 2287, 64091, 4932, 4896, and 6035. Text size in VRML2 object is small not to interfere with other VRML2 objects. One can easily zoom in and read annotations from 3DPE 3D layout. All 3D objects are clickable to show additional information.

In addition to a phylogenetic tree and species information, 3DPE needs orthologous group information for each gene. While in principle this orthology information could be supplied by the user (e.g. from databases such as HOMOLOGENE [16]), 3DPE cannot assume that users will always be able to supply such information, and therefore performs a standard orthology analysis using well-established algorithms. 3DPE separates paralogs into distinct orthologous groups following the simple rule that (by definition) any pair of homologs in one species must be paralogs. To decide whether genes from different species are orthologs, 3DPE currently follows the widely used conservative criterion of reciprocal best hits [11-14]. It should be noted that a different criterion would only change the Z-coordinate of genes, leaving typical views (e.g. Figure 2) large unchanged. Briefly, for each gene, its best hit in each species is identified based on minimum pairwise evolutionary distances; an orthologous group is defined as a fully connected clique of reciprocal best hits.

### Constructing 3D phylogeny layout

3DPE enforces two layout constraints: 1) genes from the same species must have the same X coordinates; 2) genes from the same orthologous group must have the same Z coordinates. The 3DPE layout problem can therefore be reduced to choosing 1) the order of species on the X axis; 2) the order of orthologous groups on the Z axis. Thus, for a dataset with *i *total species and *j *distinct orthologous groups, the number of possible 3DPE layouts is *i*!*j*!. 3DPE assesses the visual clarity of these distinct layout possibilities by a very simple criterion: it seeks to minimize the number of "edge-crossing" (where two tree branch edges cross in X or Z; for example, if closely related paralogs are separated by more distant paralogs in the layout, this will result in "edge-crossings" where one branch of the tree must cross another branch visually). In an effort to reduce edge-crossings, 3DPE employs several new approaches. First, it reorients 2D phylogenetic trees by evolutionary order relative to the tree's root; specifically, depth-first search (DFS, starting from root) preferentially descending the shorter child branch first at each node. The algorithm visits all internal nodes one by one and decides the orientation of each child by two simple rules: deepest subtree first (tree depth is defined as number of edges from leaf to root node) and largest subtree first (defined as the total number of leaves in given subtree). By convention, 3DPE lays out the tree in a consistent orientation based on tree depth in descending order: genes with maximum tree depth values (furthest from root) at left front corner and genes with minimum tree depth values (closest to root) at right back corner of 3D layout. Second, while walking the reoriented tree via DFS traversal in post-order, initial coordinates are assigned to species (X) and orthologous groups (Z); as each gene is visited, if its species does not yet have a coordinate, it is assigned the next X value (X+1), and similarly for its orthologous group (on Z). When available, pairwise distances from PROTDIST were used to determine the distances between species or orthologous groups. Third, 3DPE minimizes any remaining simple edge-crossings using several heuristic rules by switching coordinates of two species or orthologous groups [17]. When edge-crossings cannot be eliminated by any branch orientation swap, this is a useful indicator of possible lateral gene transfer events (see Fig. 1 for an example). Finally, X and Z coordinates for each internal node are calculated as the averages of its two child nodes, Y coordinates as evolutionary distance from original phylogenetic trees. 3DPE uses Scientific Python with extensions to output its 3D visualizations in the standard VRML2 format (viewable in any VRML2 viewer or web browser plugin); other 3D file formats could easily be generated.

## Implementation

### 3DPE analysis of the COG database

As a demonstration of the value of 3DPE for interpreting complex phylogenies, and as a resource for the microbial genome research community, we have performed 3DPE analysis of the complete Clusters of Orthologous Genes (COG) database of microbial genomes. We downloaded the COG database (2003 release) from NCBI and all flat files were parsed and stored in MySQL database. We next constructed 2D phylogenetic trees using standard methods: CLUSTALW [18] to generate multiple alignments, which are input files for PHYLIP packages [19]; PROTDIST and NEIGHBOR (UPGMA) from PHYLIP packages with default option were used to generate traditional 2D phylogenetic trees. The UPGMA method in NEIGHBOR assumes molecular clock and generates a rooted tree [19]. 3DPE used pairwise distance from PROTDIST to assign the distances between species or orthologous groups. Pre-calculated CLUSTALW multiple alignments and PHYLIP trees were also stored in MySQL for fast processing.

3DPE currently has several limitations: 1) It requires a rooted tree as input. In general, it is recommended that users employ outgroup analysis to identify the likely root location of their input tree [20]. 2) It requires branch lengths to be provided for the tree, and currently expects these to conform to the molecular clock assumption (although we expect to relax this expectation in the future). 3) The pre-calculated trees for the COG database viewable on the 3DPE website were generated using UPGMA. We encourage users to employ their preferred tree construction methods (such as Neighbor-Joining) for generating input trees to view in 3DPE.

### VRML2 viewer & plugin

VRML2 is a well-known standard format for 3D visualization. X3D has been developed as a more extensible successor to VRML2, but many VRML2 viewer and plugin are available publicly. However, most of programs are focused on visualizing a single large VRML2 file running in local machine. Because implementation of interactive web-based application is dependent on availability of VRML2 plugin, we used the Cortona Plugin for developing 3DPE as a web-based application and it is successfully tested on Firefox and Microsoft Internet Explorer running in Microsoft Windows. Popup blocking in the web browser must be disabled to use all of its functionality; alternatively one can add the 3DPE web server to the browser's list of allowed hosts. Otherwise, some of 3DPE interactive features cannot be viewed; in some cases this can crash the web browser. A basic tutorial for Cortona Plugin is available at the 3DPE web site.

### Interactive web interface and user tree format

We used simple Python CGI to construct interactive interface and requested data will be retrieved from MySQL database based on user queries for fast processing. Moreover, 3DPE basic view can be manipulated in several ways. First, it can be zoomed, panned or rotated in 3D in any web browser (features of VRML2 Cortona Plugin). Text size in VRML2 object is small not to interfere with other VRML2 objects and one can zoom in & out to read annotations. Second, users can click any item for more information: gene information (LocusTag, GenBank GI linking to NCBI protein database, species and sequence, by clicking the gene); multiple alignment; a protein family summary (by clicking the root of the tree); subtree highlighting (clicking any edge highlights all edges below it in the tree); subtree view (clicking any node reduces the view to just the subtree below that node); connectivity highlighting (clicking any two edges highlights the path connecting them). Users can upload any phylogenetic tree for viewing in 3DPE using modified version of Newick formats (see Methods), online at 3DPE website. To demonstrate the utility of 3DPE, we have also pre-computed a database of phylogenetic trees for all proteins in the COG microbial sequence database, available online as live 3DPE views. Users can search the database using COG ID and text searches against PubMed records.

## Results and Discussion

### Interpreting 3D phylogeny

3DPE is a novel web-based tree viewer that makes it easy to distinguish gene duplication events (paralog branching) from speciation branching. It projects gene phylogeny onto three spatial axes: each species is mapped to a unique location on the X-axis; each orthologous group is assigned a unique plane on the Z-axis; and the Y-axis represents evolutionary distance (Fig. 2). Genes are shown as boxes at the bottom, colored by species. This layout makes it trivially easy to perceive paralogs, which traditionally can require some scrutiny in a large tree. It also becomes easy to distinguish speciation branchings (horizontal, X-axis) vs. paralog branchings (depth, Z-axis). For example, in COG1222 we can see that the olive (64091, *Halobacterium sp. NRC-1*) paralogs were created subsequent to the green (188937, *Methanosarcina acetivorans C2A*) speciations, whereas the yellow-red (6035, *Encephalitozoon cuniculi*) paralogs branched before these species diverged.

3DPE also provides a valuable tool for cross-validating different aspects of the tree, and checking common phylogenetic assumptions such as the molecular clock principle. 3DPE's spatial layout makes it easy to see whether different paralog subtrees are consistent. For example, in COG1222, the yellow-red paralogs all show the same speciation trees (Fig. 2). The consistent spatial layout also makes it possible to spot anomalies such as possible horizontal gene transfer or molecular clock violations at a glance. For example, edges that cross subtree boundaries (such as the olive to green connection in COG1222) suggests that either the tree is incorrect or a horizontal gene transfer event has occurred. Similarly, variations in the lengths of connections between subtrees can suggest violations of the molecular clock assumption. For example, in COG1222, the last paralog subtree (at the back of the Z axis) shows a much larger evolutionary distance between the yellow vs. orange (4896, *Schizosaccharomyces pombe*) species, compared with the other paralog subtrees. Thus a violation of the molecular clock assumption appears to have occurred. Of course, while 3DPE can provide clues as to whether a molecular clock violation can be suspected, users should test such hypotheses rigorously using other methods [21]. In general, it is recommended that users employ outgroup analysis to identify the likely root location of their input tree [20].

**Figure 3:**
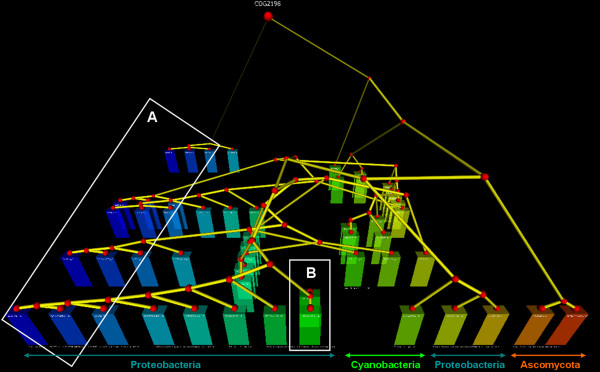
**3DPE main view mode for COG2198, FOG: HPt domain**. Species (Taxonomy IDs): 155864, 83334, 83333, 99287, 632, 666, 747, 71421, 103690, 1148, 287, 381, 4932, and 4896.

Figure 3 shows a simple example of how to distinguish recent duplications from more ancient paralogs, based on their order relative to speciation events. On the one hand, some duplications are highly similar to each other and appear to have occurred subsequent to any speciation event, because they are only found in one species and not in other species (Figure 3, example marked B). On the other hand, other duplications are found in parallel in a series species, indicating that they were created prior to the divergence of these species (Figure 3, example marked A).

### Limitations

Although 3DPE provides powerful tools for looking at complex phylogenies, extremely large phylogenetic trees can still be too much to interpret in a single 3DPE view. As a partial solution, the 3DPE web interface makes it easy to select any subtree for extraction as a separate 3DPE view. This enables the user to browse different parts of a huge tree easily and without information loss. Another limitation is the availability and ease of use of 3D viewing software. Although we have made 3DPE compatible with the well-known VRML2 standard for viewing 3D data using web browser plugins, we feel current VRML2 viewers are not completely satisfactory in their availability and ease of use. For example, while a number of VRML2 viewer programs are available (Cortona, FreeWRL, etc.), certain platforms do not yet have plugin support (e.g. new Macintoshes using Intel processors). Furthermore, current plugins are not as easy to use as we would like; e.g. the user must learn a particular plugin's mouse and keyboard commands for 3D navigation. However, VRML2 plugins are rapidly improving, and are likely to be widely available and easier to use in the future. In order to maintain up-to-date contents, we will update 3DPE if new COG database is released. Furthermore, we will support other types of 3D viewer & plugin for various users working in various platforms.

## Availability and requirements

Project name: 3DPE (3D Phylogeny Explorer)

Project homepage: 

Operating system(s): Microsoft Windows, Mac OSX

Programming Language: Python, VRML2

Other requirements: Cortona VRML2 Plugin (Mac OSX version is incomplete, some of VRML2 features may not be viewable)

License: Free, available at 3DPE website

## Authors' contributions

NK developed the method, constructed database and website, and wrote the manuscript. CL planned the research and revised this manuscript. All authors have read and approved the final manuscript.
